# Multiplex quantitative analysis of cancer-associated fibroblasts and immunotherapy outcome in metastatic melanoma

**DOI:** 10.1186/s40425-019-0675-0

**Published:** 2019-07-23

**Authors:** Pok Fai Wong, Wei Wei, Swati Gupta, James W. Smithy, Daniel Zelterman, Harriet M. Kluger, David L. Rimm

**Affiliations:** 1 Department of Pathology, Yale School of Medicine, New Haven, CT 06510 USA; 2 Yale Cancer Center, Yale School of Medicine, New Haven, CT 06510 USA; 3 Department of Biostatistics, Yale School of Public Health, New Haven, CT 06510 USA; 4 Section of Medical Oncology, Department of Internal Medicine, Yale School of Medicine, New Haven, CT 06510 USA; 5 Department of Pathology, Yale School of Medicine, 310 Cedar St, BML 116, PO Box 208023, New Haven, CT 06520 USA

**Keywords:** Biomarkers, Cancer-associated fibroblasts, Fibroblast activation protein, Immunotherapy, Melanoma, PD-1

## Abstract

**Background:**

The cancer-associated fibroblast (CAF) population is implicated in immune dysregulation. Here, we test the hypothesis that CAF profiles in pretreatment tumor specimens are associated with response to immune checkpoint blockade of programmed cell death 1 (PD-1).

**Methods:**

Pretreatment whole tissue sections from 117 melanoma patients treated with anti-PD-1 therapy were assessed by multiplex immunofluorescence to detect CAFs defined by Thy1, smooth muscle actin (SMA), and fibroblast activation protein (FAP). Two independent image analysis technologies were used: inForm software (PerkinElmer) to quantify cell counts, and AQUA™ to measure protein by quantitative immunofluorescence (QIF). CAF parameters by both methodologies were assessed for association with previously measured immune markers (CD3, CD4, CD8, CD20, CD68, PD-L1), best overall response, progression-free survival (PFS), and overall survival (OS).

**Results:**

CAF parameters, by cell counts or QIF, did not correlate with immune markers nor with best overall response. However, both Thy1 and FAP cell counts had significant positive associations with PFS (all *P* < 0.05) and OS (all *P* < 0.003). SMA cell counts showed negative associations with outcome in anti-PD-1 treated patients. Similar associations were not observed in a control cohort of historical melanoma patients predating immunotherapy. Instead, FAP was a negative prognostic biomarker (*P* = 0.01) in the absence of immunotherapy. Multivariable analyses revealed significant PFS and OS associations with the CAF parameters were independent of baseline variables.

**Conclusions:**

Pretreatment CAF profiles are associated with melanoma immunotherapy outcome. Multiplex CAF analysis has potential as an objective companion diagnostic in immuno-oncology.

**Electronic supplementary material:**

The online version of this article (10.1186/s40425-019-0675-0) contains supplementary material, which is available to authorized users.

## Introduction

Immune checkpoint blockade has become a new standard in melanoma immunotherapy and the overall survival of patients with metastatic disease has improved from ~ 9 months before 2011 to greater than 3 years [1–3]. The tumor-infiltrating lymphocyte (TIL) population expresses immune checkpoints, programmed cell death 1 (PD-1), which is targeted by pembrolizumab and nivolumab; and cytotoxic T-lymphocyte associated protein 4 (CTLA-4), which is targeted by ipilimumab [4]. Nevertheless, clinical benefit is limited to ~ 40% of metastatic melanoma patients treated with anti-PD-1 therapy, which is compounded by the lack of approved predictive strategies [1, 5]. Due to widespread use of PD-1 blockade and its recent introduction into the adjuvant setting [6], there is an increasing need for robust biomarkers to inform the practice of precision immuno-oncology [7].

The cancer-associated fibroblast (CAF) population engages in a complex and poorly understood interplay with tumor cells and immune cells, and are the predominant stromal cell type within the tumor microenvironment. CAFs are characterized by expression of Thy1, with subsets expressing smooth muscle actin (SMA) or fibroblast activation protein (FAP) [8, 9]. Thy1 is a glycophosphatidylinositol (GPI)-anchored cell surface protein that binds to integrins and may be involved in cell–cell adhesion [10]. SMA is a major component of the contractile apparatus that allows fibroblasts to produce contractile force [11]. FAP is a type II transmembrane serine protease that cleaves collagen I as an endopeptidase and engages in post-translational modification of neuropeptide Y as a dipeptidyl peptidase, which is the rare ability to hydrolyze the post-proline bond two residues from the N-terminus of substrates [12]. FAP is weakly expressed or not detected in normal adult tissues but is upregulated at sites of activated stroma in tumors and in chronic inflammation [13]. Emerging preclinical evidence implicates CAFs in immune dysregulation and response to immunotherapy [14–16]. However, CAFs represent a heterogeneous group and different CAF subsets may have opposing functions. A more comprehensive understanding of different CAF subsets as well as their impact on human immunotherapy outcome is needed.

We hypothesized that pretreatment CAF profiles of patient tumors would be associated with immunotherapy outcome. However, predictive biomarkers strictly require statistical evidence from a formal test for interaction in randomized placebo-controlled studies, which are no longer ethically possible for melanoma. Therefore, we tested a control cohort of historical melanoma patients predating immunotherapy instead to distinguish prognostic value and show a specific association between the biomarker and treatment outcome. We describe this type of biomarker as “indicative”, a separate category from truly predictive biomarkers under existing statistical definitions [17]. Briefly, indicative value is demonstrated when: [1] the hazard ratio is statistically significant in the treatment cohort and is not significant in the control cohort; or [2] the hazard ratio is statistically significant in both the treatment and control cohorts, but the respective 95% confidence intervals do not significantly overlap. The former characteristic is purely indicative, and the latter is both prognostic and indicative [17].

Here, we assess the clinical significance of CAFs for the prediction of immunotherapy outcome in metastatic melanoma. We hypothesize that the expression of these candidate biomarkers, Thy1, SMA, and FAP, will classify anti-PD-1 therapy treated patients into groups that benefit and those that do not.

## Methods

### Patient cohort

The study cohort is a retrospective collection of 117 melanoma patients treated with anti-PD-1 therapy in the metastatic setting between 2011 and 2017 at Yale Cancer Center. Uveal melanoma was excluded [18]. The analysis only included pretreatment formalin-fixed, paraffin-embedded (FFPE) specimens after review by a board-certified pathologist. All specimens were collected from the Yale Pathology archives. Clinicopathological data were collected from clinical records and pathology reports; the data cut-off date was September 1, 2017. Response Evaluation Criteria in Solid Tumors (RECIST) 1.1 were used to determine best overall response as complete response (CR), partial response (PR), stable disease (SD), or progressive disease (PD), and objective response rate (ORR; CR/PR) and disease control rate (DCR; CR/PR/SD) [19]. A historical cohort of 194 melanoma patients, collected prior to the advent of anti-PD-1, was used as the control group. Cohort characteristics are detailed in Table 1. Other characteristics of the anti-PD-1 treated cohort including the melanoma specimen, time interval to anti-PD-1 therapy, and prior immune checkpoint blockade are shown in Additional file 1: Table S1. All patients provided written informed consent or waiver of consent. The study was approved by the Yale Human Investigation Committee protocol #9505008219 and conducted in accordance with the Declaration of Helsinki.

**Table 1:** Clinicopathological characteristics of the melanoma cohort treated with anti-PD-1 therapy and the control melanoma cohort for CAF profiling

Characteristic	Anti-PD-1 patients, No. (%)	Objective response rate (CR/PR), No. (%)	Disease control rate (CR/PR/SD), No. (%)	Control patients, No. (%)
Overall	117 (100)	55 (47)	81 (69)	194 (100)
Age (y)				
< 65	67 (57)	34 (51)	51 (76)	87 (45)
≥ 65	50 (43)	21 (42)	30 (60)	107 (55)
Sex				
Male	70 (60)	35 (50)	48 (69)	110 (57)
Female	47 (40)	20 (43)	33 (70)	84 (43)
Treatment				
Pembrolizumab	41 (35)	20 (49)	30 (73)	0
Nivolumab	18 (15)	7 (39)	9 (50)	0
Ipilimumab plus nivolumab	58 (50)	28 (48)	42 (72)	0
Prior immune checkpoint blockade				
Yes	36 (31)	13 (36)	22 (61)	0
No	81 (69)	42 (52)	59 (73)	194 (100)
Mutation status				
BRAF	39 (33)	19 (49)	27 (69)	NA
NRAS	18 (15)	8 (44)	11 (61)	NA
KIT	2 (2)	1 (50)	2 (100)	NA
None detected	58 (50)	27 (47)	41 (71)	NA
Stage at diagnosis				
I	24 (21)	14 (58)	19 (79)	77 (40)
II	23 (20)	12 (52)	16 (70)	80 (41)
III	38 (32)	16 (42)	24 (63)	30 (15)
IV	20 (17)	6 (30)	13 (65)	3 (2)
Not available	12 (10)	7 (58)	9 (75)	4 (2)

Abbreviations: *CAF* cancer-associated fibroblast, *CR* complete response, *NA* not available, *PR* partial response, *SD* stable disease

### Multiplex immunofluorescence CAF panel

5-plex immunofluorescence using isotype-specific antibodies was performed on FFPE whole tissue sections for simultaneous detection of markers as previously described [20]. The protocol is detailed in the Additional file 1.

### Image analysis by two independent methods: cell counts versus quantitative immunofluorescence

Cell counts were determined by the pattern recognition software, inForm Tissue Finder (PerkinElmer, Waltham, MA, USA), on multispectral images acquired using a Vectra 3 system (PerkinElmer) as previously described [21]. Multispectral images were decomposed into their various components by spectral unmixing using a digital spectral library consisting of spectral profiles of each of the fluorophores. Automated tissue segmentation identified tumor and stroma regions. Cell segmentation within these regions identified individual cells and respective nuclei, cytoplasm, and membrane components using signal in the nucleus and membrane as internal and external cell borders, then cells were phenotyped for marker expression. Cell counts for each melanoma case were calculated in terms of the number of cells positive for the marker of interest as a percentage of the cell population in which it was measured. Protein expression of the various markers was determined by the automated quantitative analysis (AQUA) method of QIF on fluorescence images acquired using a PM-2000 system (Navigate BioPharma, Carlsbad, CA, USA) as previously described [22]. A total compartment, consisting of all cells, or a Thy1 compartment was generated by automated processing and thresholding of the DAPI signal or Thy1 signal, respectively. QIF scores were calculated by dividing the summed pixel intensities for the marker of interest by the area of the compartment in which it was measured [22]. Overall QIF scores were derived for each melanoma case by averaging scores from each field of view.

### Statistical analysis

Statistical comparisons for cell count and QIF data were made using unpaired *t*-test or analysis of variance (ANOVA) followed by Tukey’s test for multiple comparisons as appropriate. The Lausen and Schumacher method of maximally selected rank statistics, a powerful nonparametric method for assessing predictive power of a continuous variable for a dependent variable, was used to determine thresholds to objectively define low and high statuses for the measured CAF parameters [23]. Kaplan–Meier estimates of progression-free survival (PFS) and overall survival (OS) functions were computed and comparisons were made by the log-rank test. Multivariable Cox proportional hazards models included age, sex, mutation status, stage, treatment, and prior immune checkpoint blockade as covariates [24–27]. All statistical tests were two-sided and statistical significance was defined as *P* < 0.05. Statistical analysis was performed using GraphPad Prism 7 (GraphPad Software, La Jolla, CA, USA) and JMP Pro 13 (SAS Institute, Cary, NC, USA). The sample size of 117 patients had at least 80% power at *P* = 0.05 to detect a difference in means of 0.52 standard deviations in each CAF parameter for responders (CR/PR) versus non-responders (SD/PD).

## Results

### Correlation between cell counts and quantitative immunofluorescence

Tissue biomarkers can be quantified in situ by counting positive cells for the biomarker or in terms of quantitative protein expression levels per unit area. These are two independent types of parameters and may be nonequivalent in clinical significance. The relationship between cell counts and QIF was assessed by linear regression, which revealed a positive correlation for Thy1 (R^2^ = 0.35), SMA (R^2^ = 0.36), and FAP (R^2^ = 0.62) (Additional file 1: Figure S1A). On the contrary, there was no correlation between different markers, which confirmed their independence (Additional file 1: Figure S1B).

### Immune markers and CAF parameters

**Fig. 1:**
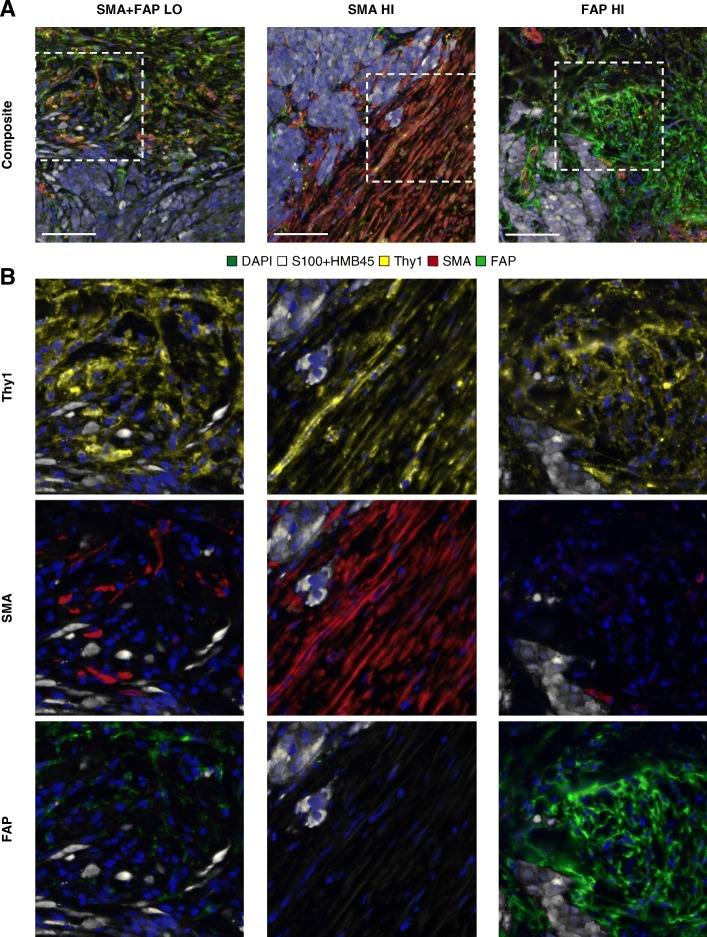
Cancer-associated fibroblast profiling by multiplex immunofluorescence in melanoma. Representative multispectral immunofluorescence images of CAF (Thy1, SMA, FAP) profiling in melanoma (magnification × 200; scale bar = 100 μm) (**a**), and corresponding visualizations of each CAF marker with nuclei (DAPI) and melanoma cells (S100 and HMB45) for the regions indicated (**b**). Abbreviations: CAF, cancer-associated fibroblast; DAPI, 4′,6-diamidino-2-phenylindole; HI, high; LO, low

**Fig. 2:**
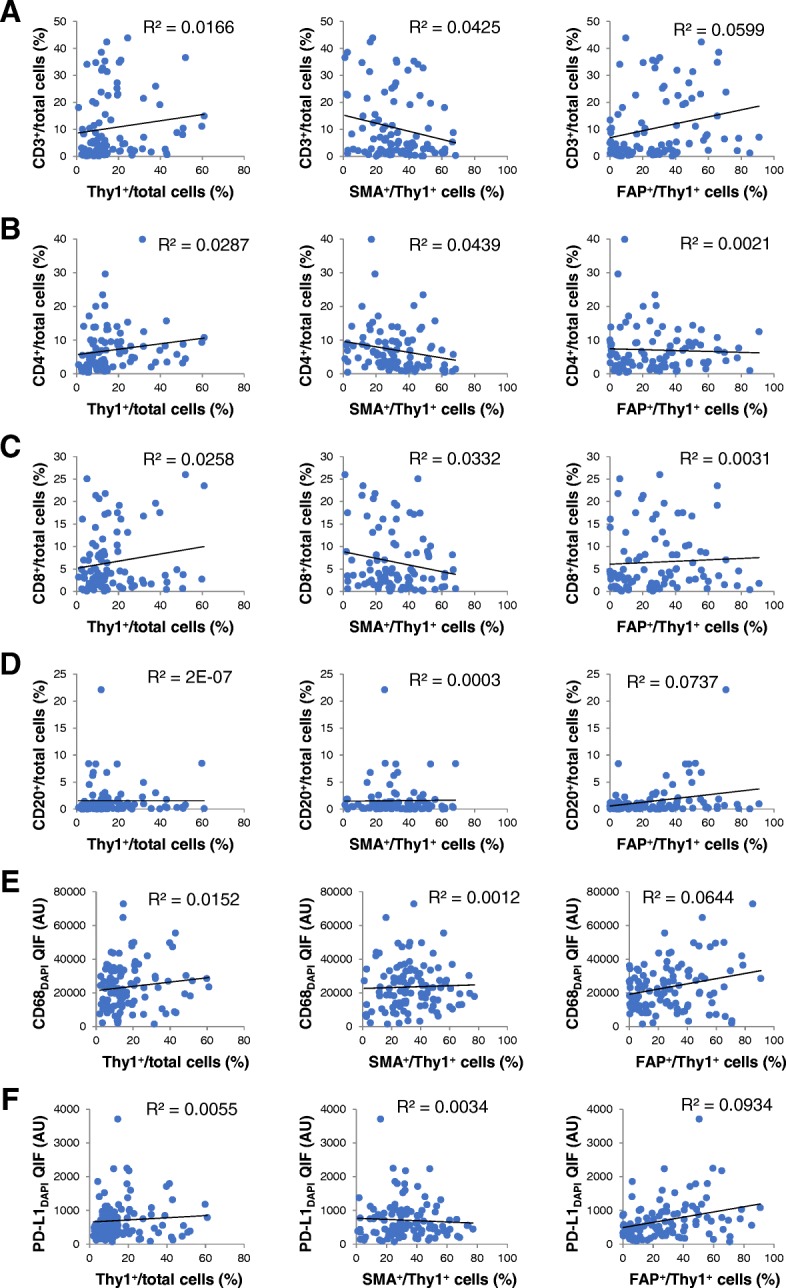
Immune markers and CAF parameters by cell counts in melanoma. Relationships between CAF (Thy1, SMA, FAP) markers and CD3 (**a**), CD4 (**b**), CD8 (**c**), CD20 (**d**), CD68 (**e**) and PD-L1 (**f**) in melanoma. Abbreviations: AU, arbitrary units; CAF, cancer-associated fibroblast; QIF, quantitative immunofluorescence

Pretreatment whole tissue sections from 117 melanoma patients treated with anti-PD-1 therapy underwent CAF (Thy1, SMA, FAP) profiling by multiplex immunofluorescence (Fig. 1). The relationship between CAFs and infiltration of immune cell populations or expression of immune markers in melanoma was assessed by linear regression with previous data [17]. There was no correlation between the CAF parameters and CD3, CD4, CD8, CD20, CD68, or PD-L1, which confirmed their independence of those immune markers (Fig. 2 and Additional file 1: Figure S2).

### Best overall response by RECIST and CAF parameters

The CAF parameters, by cell counts or QIF, were analyzed in relation to specimen-specific variables and tumor burden classifications defined by RECIST 1.1 [19]. There were no significant associations with sex or mutation status of melanoma patients for the CAF parameters by cell counts or QIF (all *P* > 0.05; Additional file 1: Figure S3). Neither Thy1, SMA, nor FAP cell counts were associated with best overall response defined by RECIST (all *P* > 0.05; Additional file 1: Figure S4A). The corresponding QIF data (Additional file 1: Figure S4B) and further analyses on ORR and DCR (Additional file 1: Figure S5) corroborated these findings and revealed a similar lack of association with RECIST.

### Survival outcome and CAF parameters

**Fig. 3:**
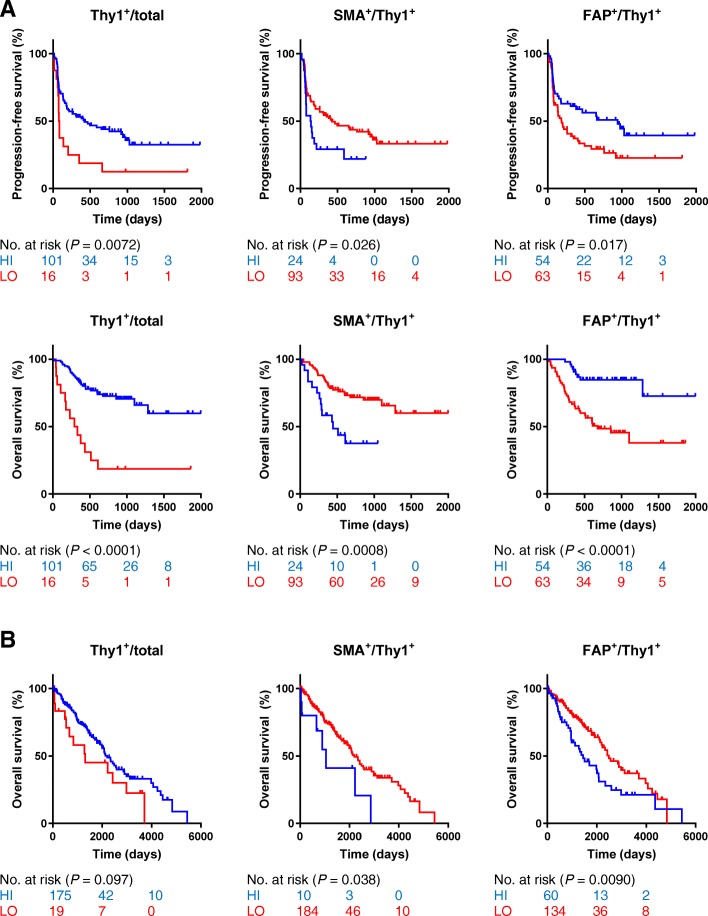
CAF parameters by cell counts and survival of melanoma patients treated with anti-PD-1 therapy and control melanoma patients. Kaplan–Meier analysis of progression-free survival and overall survival of anti-PD-1 treated melanoma patients (**a**) and overall survival of control melanoma patients (**b**) according to CAF (Thy1, SMA, FAP) parameters by cell counts. Low and high statuses were objectively defined using thresholds determined by maximally selected rank statistics (see Methods). Abbreviations: CAF, cancer-associated fibroblast; HI, high; LO, low

For survival analysis, the continuous CAF parameters were dichotomized into low and high statuses using the Lausen and Schumacher method of maximally selected rank statistics for the standardized derivation of objective thresholds from the population data (Additional file 1: Figure S6) [23]. In Cox regressions, both high Thy1 cell count and high FAP cell count were associated with prolonged PFS, whereas low SMA cell count was associated with prolonged PFS (Fig. 3a and Table 2). Similarly, OS had significant positive associations with both Thy1 and FAP cell counts, and a negative association with SMA cell count, which were specific to anti-PD-1 treated melanoma patients (all *P* < 0.003; Fig. 3a and Table 3). To determine this distinction, a control melanoma cohort predating immunotherapy with known survival outcome was assessed for prognostic value in place of a placebo arm. Similar associations were not observed in the control patients (Fig. 3b and Table 3). Remarkably, FAP cell count was a significant negative prognostic biomarker in the absence of immunotherapy (*P* = 0.01) with an inverted hazard ratio (HR = 0.57, 95% CI, 0.37–0.88) relative to that of the anti-PD-1 patients (HR = 4.11, 95% CI, 2.05–9.14) (Table 3). Multivariable analyses further revealed significant survival associations with the CAF parameters, particularly for FAP, independent of age, sex, mutation, stage, treatment, and prior immune checkpoint blockade (Tables 2–3). The QIF data showed similar trends in relation to survival (Additional file 1: Figure S7 and Additional file 1: Tables S2–S3). Survival analysis by treatment group generally showed similar trends despite the reduction in statistical power (Additional file 1: Tables S4–S5).

**Table 2:** Univariable and multivariable Cox regression analyses for progression-free survival of melanoma patients and CAF parameters by cell counts

Variable (LO/HI)	Anti-PD-1 PFS					
	Univariable analysis		Multivariable^a^ analysis per variable		Multivariable^a^ analysis with Thy1, SMA, FAP	
	HR (95% CI)	*P* value	HR (95% CI)	*P* value	HR (95% CI)	*P* value
Thy1^+^/total	2.18 (1.17–3.81)	0.016	2.34 (1.21–4.28)	0.013	1.90 (0.98–3.48)	0.058
SMA^+^/Thy1^+^	0.55 (0.32–0.97)	0.038	0.55 (0.32–0.99)	0.048	0.71 (0.40–1.31)	0.26
FAP^+^/Thy1^+^	1.77 (1.11–2.89)	0.017	2.08 (1.28–3.44)	0.0030	1.79 (1.06–3.04)	0.031

Abbreviations: *CAF* cancer-associated fibroblast, *CI* confidence interval, *HI* high, *HR* hazard ratio, *LO* low, *PFS* progression-free survival

^a^Cox proportional hazards model included age, sex, mutation status, stage, treatment, and prior immune checkpoint blockade as covariates

**Table 3:** Univariable and multivariable Cox regression analyses for overall survival of melanoma patients and CAF parameters by cell counts

Variable (LO/HI)	Control OS		Anti-PD-1 OS					
	Univariable analysis		Univariable analysis		Multivariable^a^ analysis per variable		Multivariable^a^ analysis with Thy1, SMA, FAP	
	HR (95% CI)	*P* value	HR (95% CI)	*P* value	HR (95% CI)	*P* value	HR (95% CI)	*P* value
Thy1^+^/total	1.65 (0.87–2.88)	0.12	4.66 (2.34–8.82)	< 0.0001	4.67 (2.19–9.53)	0.0001	3.02 (1.44–6.10)	0.0044
SMA^+^/Thy1^+^	0.45 (0.22–1.07)	0.070	0.34 (0.18–0.68)	0.0029	0.32 (0.16–0.67)	0.0027	0.62 (0.30–1.31)	0.20
FAP^+^/Thy1^+^	0.57 (0.37–0.88)	0.012	4.11 (2.05–9.14)	< 0.0001	4.64 (2.27–10.52)	< 0.0001	3.61 (1.65–8.56)	0.0011

Abbreviations: *CAF* cancer-associated fibroblast, *CI* confidence interval, *HI* high, *HR* hazard ratio, *LO* low, *OS* overall survival

^a^Cox proportional hazards model included age, sex, mutation status, stage, treatment, and prior immune checkpoint blockade as covariates

## Discussion

In this study, we determine the clinical significance of pretreatment CAF (Thy1, SMA, FAP) profiles according to both in situ cell counts and QIF protein expression in relation to immunotherapy outcome in metastatic melanoma. PFS and OS had positive associations with Thy1 and FAP cell counts, and negative associations with SMA cell count, which were specific to anti-PD-1 treated patients. Significant PFS and OS associations with the CAF parameters were independent of age, sex, mutation, stage, treatment, and prior immune checkpoint blockade [24–27]. While the two quantitative methods are independent, cell counts correlated with QIF and revealed concordant associations with response and survival outcome.

This study attempts to rigorously investigate multiplex CAF profiling and melanoma immunotherapy outcome, however, there are a number of limitations. The most significant limitation is the fact that predictive biomarkers strictly require statistical proof by a test for interaction in a randomized placebo-controlled trial, which is no longer ethically possible for melanoma after the approval of immune checkpoint therapy. Consequently, all post-trial predictive biomarker studies are limited by the same statistical requirement. Instead, we analyzed an anti-PD-1 treated melanoma cohort and a historical cohort predating immunotherapy to show a specific association between the biomarker and treatment outcome. Indicative value is inferred if the biomarker is associated with outcome in the treated cohort but a similar association is not observed in the control cohort. This is best demonstrated in Fig. 3 and Table 3, where the OS association with FAP undergoes a striking inversion as a function of presence or absence of anti-PD-1 therapy. Therefore, FAP has indicative value and may have future potential in a clinical assay to determine likelihood of survival benefit from anti-PD-1 therapy for melanoma. Another limitation is the fact that this is a single-institutional retrospective study with a modest sample size, even though all available relevant cases at Yale were collected at the time of the study. We look forward to prospective investigation of these assays or similar in future clinical trials, especially since PD-1 blockade is now widely used in the adjuvant setting where benefit is seen in only 1 in 5 treated melanoma patients [6]. Although our CAF profiling methodologies used quantitative fluorescence imaging systems for increased accuracy, the concept and design may be adapted for implementation on conventional pathology platforms (for example, see Hartman et al. [28]).

Recent studies indicate that mesenchymal or stromal abundance influences immunotherapy outcome [29, 30]. However, the stromal compartment is heterogeneous and different CAF subsets may have divergent effects. In the present study, the CAF population was stratified in terms of their expression of Thy1, SMA, and FAP. The differences in survival associations for SMA and FAP may reflect the functional complexity of CAF subsets. According to a single-cell RNA sequencing study, up to seven CAF subsets with unique expression phenotypes may exist in non-small cell lung cancer [9]. The identification of specific CAF subpopulations provides a foundation for future studies to deconvolute their specialized activities, which may inform the design of new diagnostic and therapeutic strategies.

The intriguing role of FAP as a negative prognostic and positive indicative biomarker in melanoma is demonstrated by its positive association with survival outcome of anti-PD-1 treated melanoma patients, and its inverse association with prognosis in the absence of immunotherapy. This is reminiscent of the well-known behavior of HER2 as a negative prognostic and positive predictive biomarker in breast cancer. Whereas HER2 is the therapeutic target in the case of breast cancer, the role of FAP in immunotherapy is not well understood. The specific association of FAP with anti-PD-1 survival advantage suggests mechanistic involvement. Recent supporting evidence has been published showing direct interactions between CAFs and T cells, mediated through coincident upregulation and engagement of PD-1 on T cells, to drive T cell dysfunction and death within tumors [31]. This CAF-mediated mechanism may explain the observed associations with survival benefit in anti-PD-1 therapy, and poor prognosis in the absence of immunotherapy. Furthermore, our data demonstrate that these biomarkers are associated with survival outcome but not RECIST-based response, which are different clinical endpoints. Multivariable analyses provided unique insights including the non-redundant role of FAP in the observed outcome associations when Thy1 and/or SMA are also included in the Cox models. Melanoma mutation status was not associated with any CAF parameter [32]. The CAF parameters also did not correlate with immune markers, which indicates independence of those measurement variables and non-redundancy, and may therefore be complementary to existing biomarkers such as CD8 and PD-L1 [33, 34]. A combination biomarker strategy is being studied to determine if combinations of CAF parameters with immune cell parameters have stronger associations with immunotherapy outcome. A predictive signature classifier computed from all available tissue data is also under consideration.

The use of two independent image analysis technologies to assess biomarkers and the concordant results from cell counts and QIF adds confidence in the findings. The AQUA method of QIF measures protein expression as cumulative signal intensity per unit compartment area, and it has been shown to be proportional to analyte concentration [35]. This is fundamentally different from counts of digitally phenotyped cells [36]. The similar results of the two methodologies suggest shared biological relevance. However, cell counts use intuitive absolute units and exhibited stronger associations with survival outcome than QIF, therefore, it may have a greater potential for clinical translation in digital precision immuno-oncology.

In summary, this study demonstrates that pretreatment CAF profiles, by in situ cell counts or QIF protein expression, are independently associated with melanoma immunotherapy outcome. The finding that FAP is a negative prognostic but positive indicative biomarker suggests mechanistic involvement and warrants further study. Multiplex CAF profiling has the potential for application as a companion diagnostic in digital precision immuno-oncology and may be complementary to existing immune-related markers for patient stratification.

## Conclusions

This study examines the clinical significance of cancer-associated fibroblast (Thy1, SMA, FAP) profiles in pretreatment tumor specimens to determine their association with immunotherapy outcome in melanoma. We find that FAP, by both digital cell counts and quantitative immunofluorescence of protein expression, shows significant positive associations with survival outcome. The positive association is independent of baseline variables in multivariable analyses. In contrast, FAP is inversely associated with prognosis in the absence of immunotherapy in a historical cohort. The novel discovery that FAP is a negative prognostic and positive indicative biomarker in melanoma suggests mechanistic involvement in anti-PD-1 survival advantage. Its independence from previously described biomarkers like CD8 and PD-L1 suggest it could have value in combination with those markers to more accurately predict outcome to immunotherapy.

## Additional file

Additional file 1: Supplementary Figure 1. Linear regressions of CAF parameters in melanoma by cell counts and quantitative immunofluorescence. Correlation between cell counts and QIF scores for CAF (Thy1, SMA, FAP) markers (A). Relationships between Thy1, SMA, and FAP by cell counts and QIF (B). Abbreviations: AU, arbitrary units; CAF, cancerassociated fibroblast; QIF, quantitative immunofluorescence. (PDF 439 kb)

## Data Availability

De-identified datasets used and/or analyzed during the current study are available from the corresponding author upon reasonable request.
