# Supplementary material for: Multiplex quantitative analysis of cancer-associated fibroblasts and immunotherapy outcome in metastatic melanoma
Source: J Immunother Cancer. 2019 Jul 23;7:194. doi: 10.1186/s40425-019-0675-0 (PMC6651990; doi:10.1186/s40425-019-0675-0)
Supplement: Supplementary file 1 — Supplementary Figure 1. Linear regressions of CAF parameters in melanoma by cell counts and quantitative immunofluorescence. Correlation between cell counts and QIF scores for CAF (Thy1, SMA, FAP) markers (A). Relationships between Thy1, SMA, and FAP by cell counts and QIF (B). Abbreviations: AU, arbitrary units; CAF, cancerassociated fibroblast; QIF, quantitative immunofluorescence. (PDF 439 kb) [file 40425_2019_675_MOESM1_ESM.pdf]

Supplementary Methods

Multiplex immunofluorescence CAF panel

Sections underwent deparaffinization at 60 °C for 30 min followed by xylene washes, then rehydration in an ethanol series. Antigen retrieval was performed in 1 mM EDTA (pH 8) at 97 °C for 20 min in a Lab Vision PT Module (Thermo Scientific, Waltham, MA, USA). Endogenous peroxidases were blocked with 2.5% hydrogen peroxide in methanol for 30 min. The following steps employed a Lab Vision Autostainer 720 (Thermo Scientific). Non-specific antigens were blocked with 0.3% BSA in TBST for 30 min. Primary monoclonal antibodies against Thy1 (1:10000; 7E1B11; Abcam, Cambridge, MA, USA), SMA (1:500; 1A4; Dako, Carpinteria, CA, USA), and FAP (1:500; EPR20021; Abcam) for CAF profiling were co-incubated at room temperature for 1 h. Sections were incubated sequentially with three horseradish peroxidase (HRP)-conjugated secondary antibodies at room temperature for 1 h before tyramide-based labelling for 10 min, followed by 1 mM benzoic hydrazide with 0.15% hydrogen peroxide for 10 min twice to quench HRP activity. The secondary antibodies were anti-rabbit EnVision reagent (Dako), anti-mouse IgG1 (1:100; eBioscience, San Diego, CA, USA), and anti-mouse IgG2a (1:200; Abcam), and the substrates were biotin tyramide (1:50; PerkinElmer, Waltham, MA, USA), TSA Plus Cy3 tyramide (1:100; PerkinElmer), and Cy5 tyramide (1:50; PerkinElmer), respectively. Sections were then treated with streptavidin–Alexa Fluor 750 conjugate (1:100; Invitrogen, Carlsbad, CA, USA) for 1 h. Finally, sections were incubated with mouse anti-S100 (1:100; 15E2E2; BioGenex, Fremont, CA, USA) and HMB45 (1:100; BioGenex) then goat anti-mouse Alexa Fluor 488 (1:100; Invitrogen) for 1 h to identify melanoma cells, counterstained with 4',6-diamidino-2-phenylindole (DAPI) to visualize nuclei, and mounted with ProLong Gold Antifade (Invitrogen).

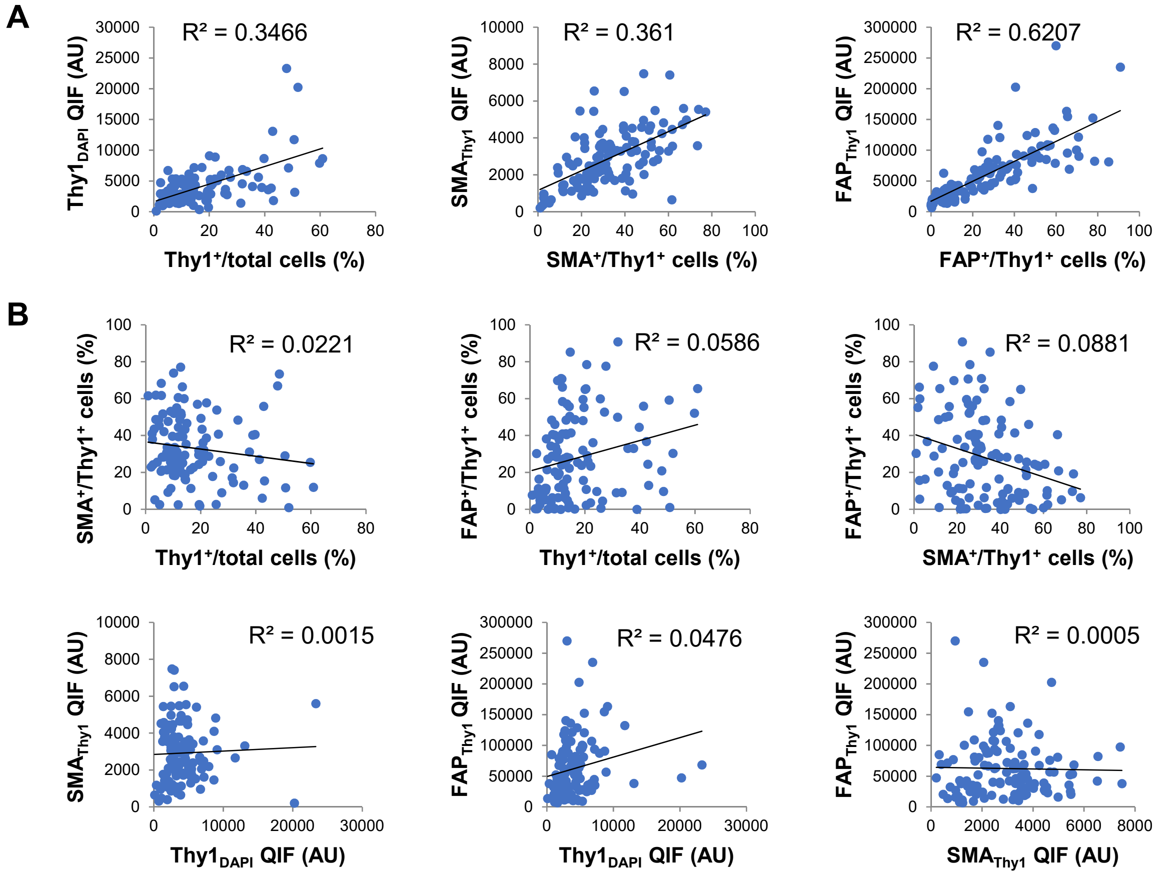

Supplementary Figure 1. Linear regressions of CAF parameters in melanoma by cell counts and quantitative immunofluorescence.
Correlation between cell counts and QIF scores for CAF (Thy1, SMA, FAP) markers (A). Relationships between Thy1, SMA, and FAP by cell counts and QIF (B). Abbreviations: AU, arbitrary units; CAF, cancer-associated fibroblast; QIF, quantitative immunofluorescence.

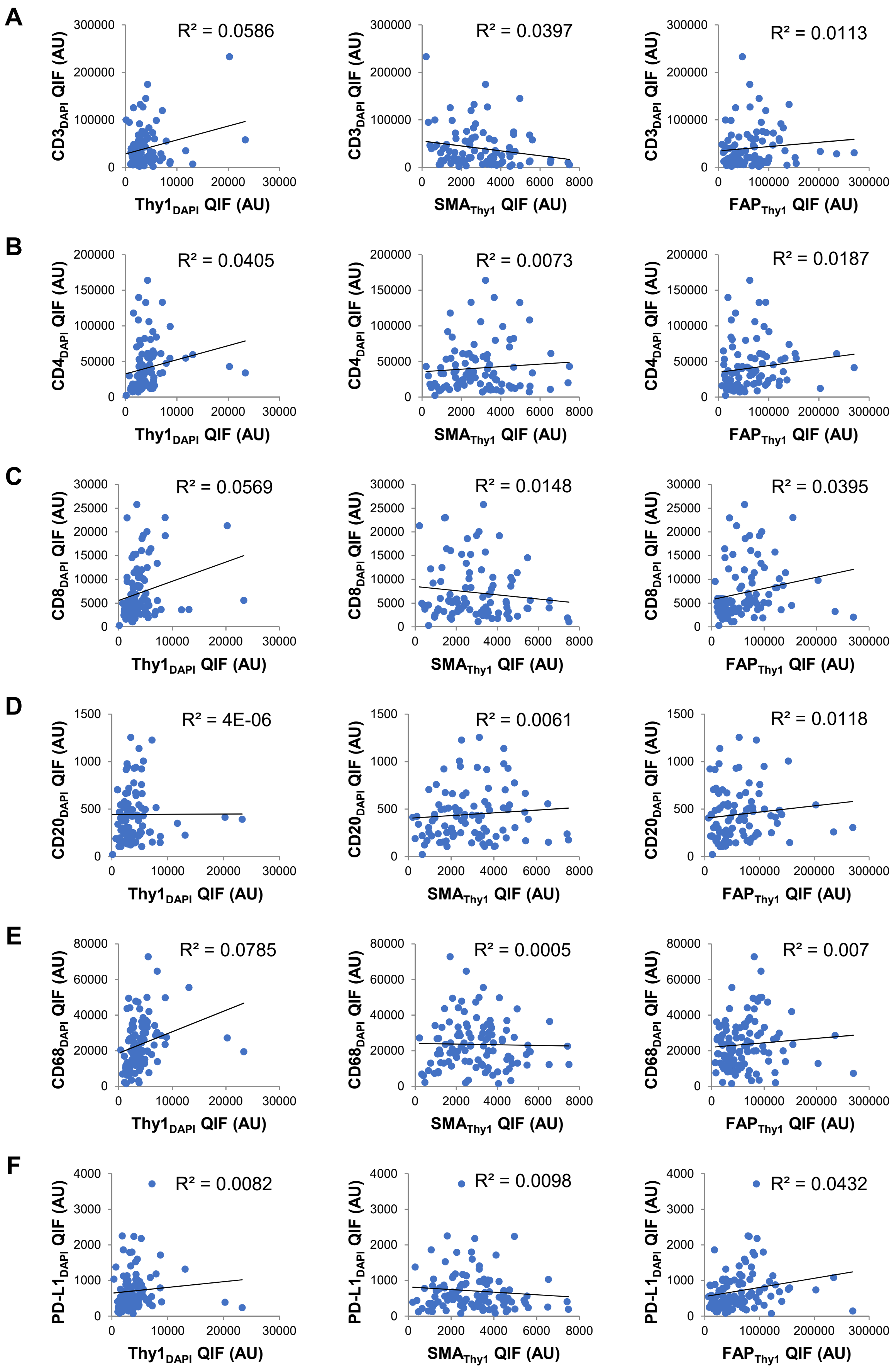
A
R² = 0.0586
R² = 0.0397
R² = 0.0113
CD3DAPI QIF (AU)
B
R² = 0.0405
R² = 0.0073
R² = 0.0187
CD4DAPI QIF (AU)
C
R² = 0.0569
R² = 0.0148
R² = 0.0395
CD8DAPI QIF (AU)
D
R² = 4E-06
R² = 0.0061
R² = 0.0118
CD20DAPI QIF (AU)
E
R² = 0.0785
R² = 0.0005
R² = 0.007
CD68DAPI QIF (AU)
F
R² = 0.0082
R² = 0.0098
R² = 0.0432
PD-L1DAPI QIF (AU)
Thy1DAPI QIF (AU)
SMAThy1 QIF (AU)
FAPThy1 QIF (AU)

Supplementary Figure 2. Immune markers and CAF parameters by quantitative immunofluorescence in melanoma.
Relationships between CAF (Thy1, SMA, FAP) markers and CD3 (A), CD4 (B), CD8 (C), CD20 (D), CD68 (E) and PD-L1 (F) in melanoma. Abbreviations: AU, arbitrary units; CAF, cancer-associated fibroblast; QIF, quantitative immunofluorescence.

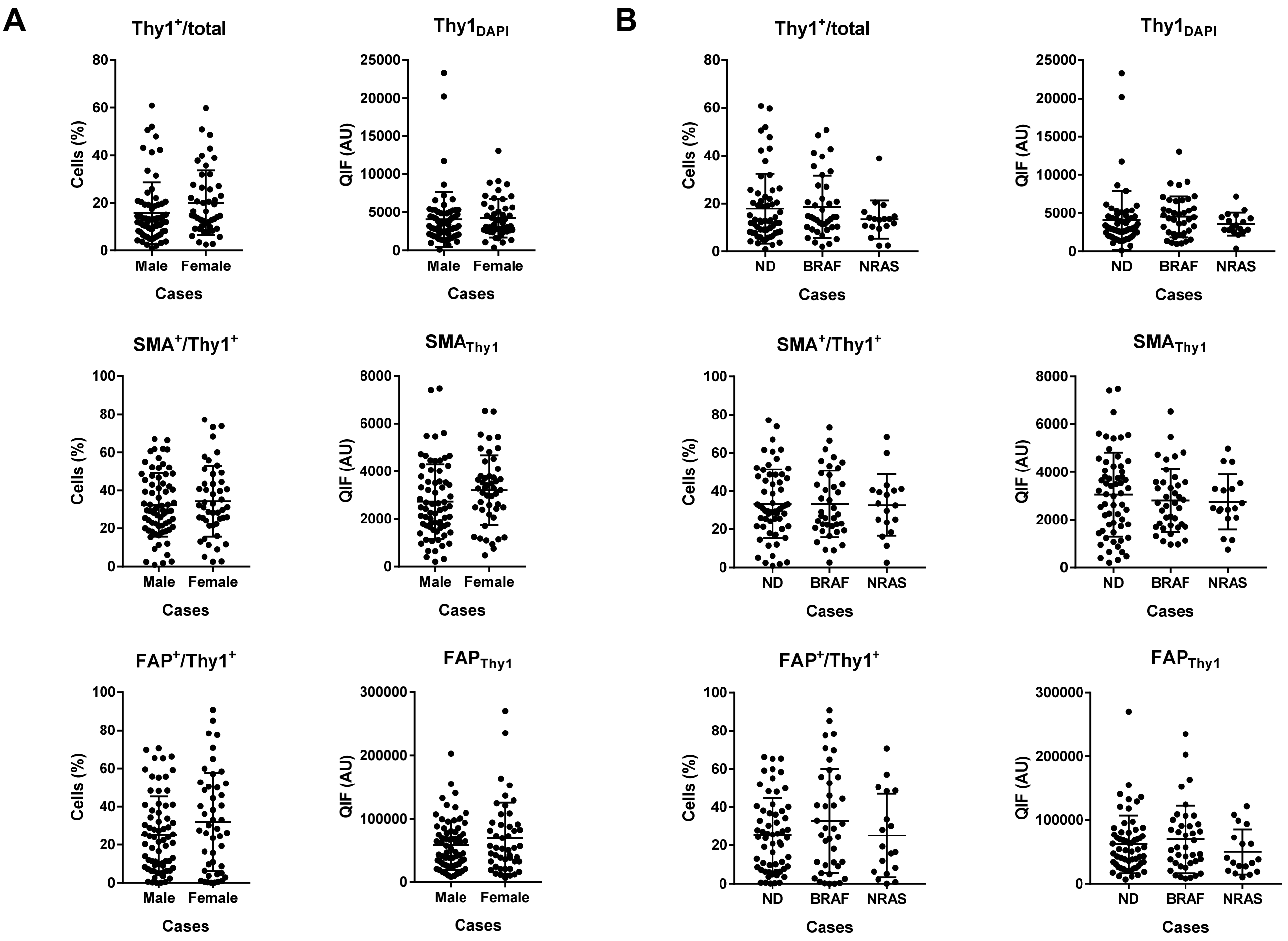

Supplementary Figure 3. Sex and mutation status of melanoma patients and CAF parameters.
CAF (Thy1, SMA, FAP) parameters by cell counts and QIF per sex (A) and mutation status (B) of melanoma patients. Data are presented as mean with standard deviation (error bars). Abbreviations: AU, arbitrary units; CAF, cancer-associated fibroblast; ND, no detection of BRAF or NRAS mutations; QIF, quantitative immunofluorescence.

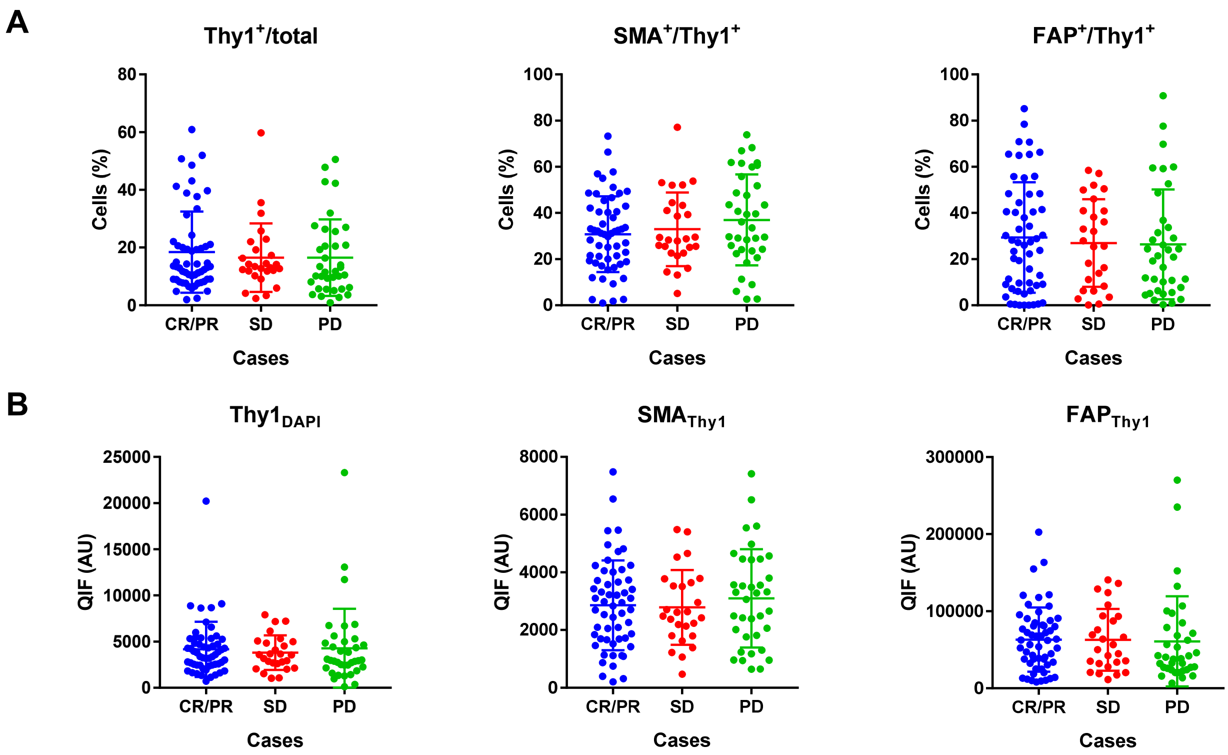

Supplementary Figure 4. RECIST categories of melanoma patients treated with anti-PD-1 therapy and CAF parameters.

CAF (Thy1, SMA, FAP) parameters by cell counts (A) and QIF (B) per RECIST categories of best overall response. Data are presented as mean with standard deviation (error bars). Abbreviations: AU, arbitrary units; CAF, cancer-associated fibroblast; CR, complete response; PD, progressive disease; PR, partial response; QIF, quantitative fluorescence; RECIST, Response Evaluation Criteria in Solid Tumors; SD, stable disease.

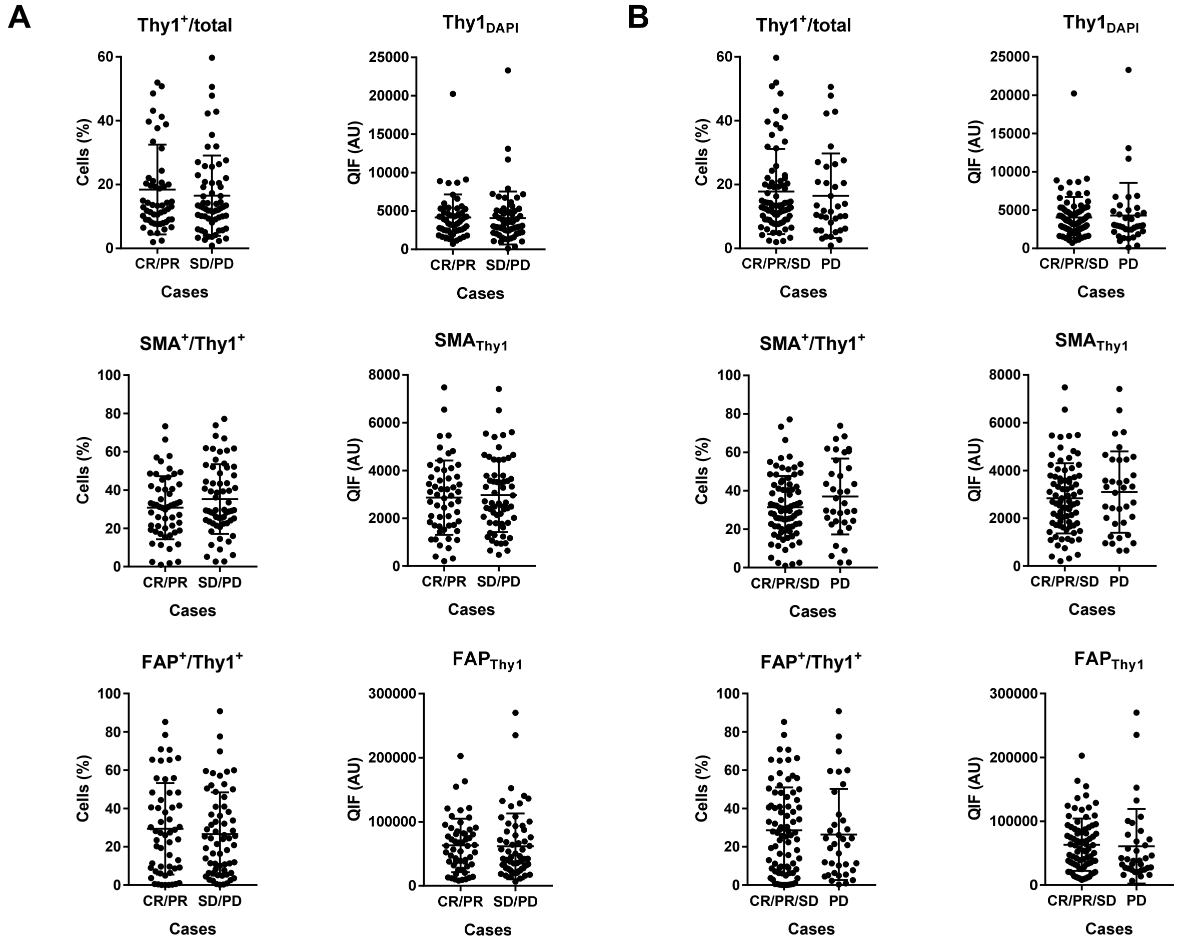

Supplementary Figure 5. Anti-PD-1 objective response rate or disease control rate and CAF parameters in melanoma patients.

CAF (Thy1, SMA, FAP) parameters by cell counts and QIF in relation to anti-PD-1 objective response rate (A) and disease control rate (B) by RECIST. Data are presented as mean with standard deviation (error bars). Abbreviations: AU, arbitrary units; CAF, cancer-associated fibroblast; CR, complete response; PD, progressive disease; PR, partial response; QIF, quantitative fluorescence; RECIST, Response Evaluation Criteria in Solid Tumors; SD, stable disease.

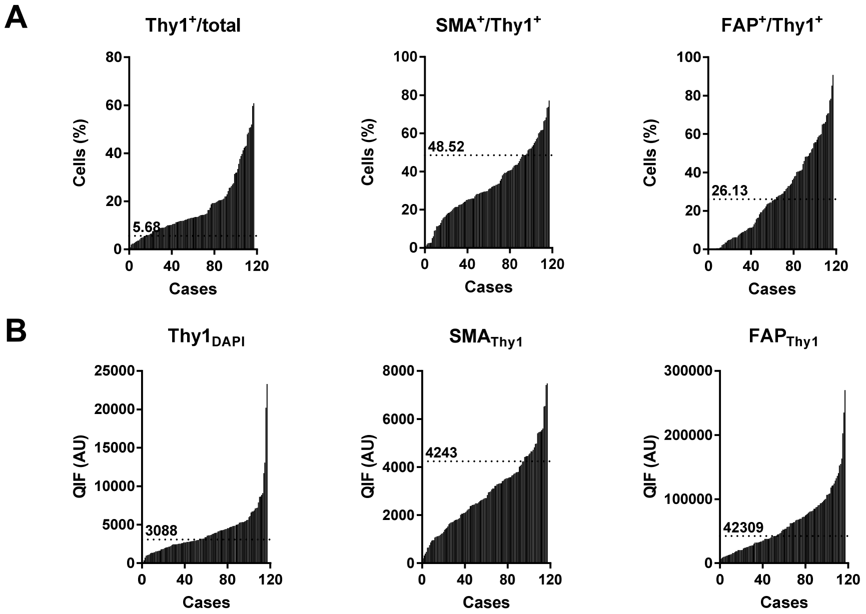

Supplementary Figure 6. Thresholds and cohort distributions of CAF parameters.
Cohort distributions of CAF (Thy1, SMA, FAP) parameters by cell counts (A) and QIF (B) with thresholds determined by maximally selected rank statistics indicated (see Methods). Abbreviations: AU, arbitrary units; CAF, cancer-associated fibroblast; QIF, quantitative immunofluorescence.

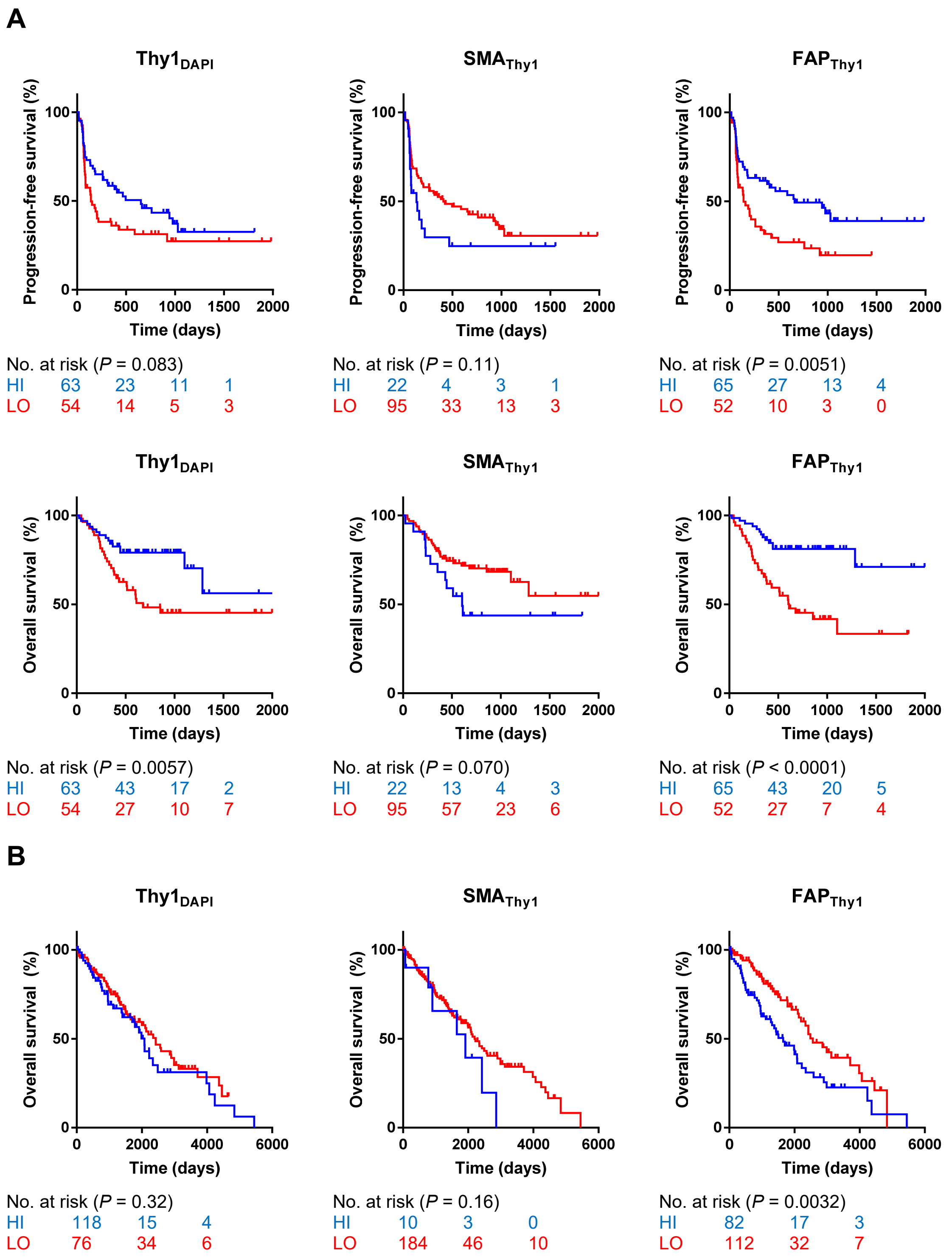

Supplementary Figure 7. CAF parameters by quantitative immunofluorescence and survival of melanoma patients treated with anti-PD-1 therapy and control melanoma patients.
Kaplan–Meier analysis of progression-free survival and overall survival of anti-PD-1 treated melanoma patients (A) and overall survival of control melanoma patients (B) according to CAF (Thy1, SMA, FAP) parameters by QIF. Low and high statuses were objectively defined using thresholds determined by maximally selected rank statistics (see Methods). Abbreviations: CAF, cancer-associated fibroblast; HI, high; LO, low; QIF, quantitative fluorescence.

Supplementary Table 1. Other characteristics of the melanoma cohort treated with anti-PD-1 therapy.

No.	Melanoma specimen	Specimen to anti-PD-1 therapy (y)	Prior immune checkpoint blockade	No.	Melanoma specimen	Specimen to anti-PD-1 therapy (y)	Prior immune checkpoint blockade
1	metastasis	≤1	ipilimumab	61	metastasis	>1	none
2	primary	≤1	none	62	metastasis	>1	nivolumab
3	metastasis	≤1	ipilimumab	63	metastasis	≤1	none
4	metastasis	≤1	none	64	primary	>1	none
5	metastasis	≤1	none	65	metastasis	≤1	ipilimumab
6	metastasis	≤1	none	66	metastasis	>1	none
7	metastasis	≤1	none	67	metastasis	>1	none
8	metastasis	≤1	ipilimumab	68	primary	>1	none
9	metastasis	>1	none	69	metastasis	>1	none
10	metastasis	>1	none	70	primary	>1	none
11	metastasis	>1	none	71	metastasis	≤1	none
12	primary	>1	none	72	metastasis	>1	none
13	metastasis	≤1	none	73	metastasis	≤1	ipilimumab
14	metastasis	>1	none	74	metastasis	>1	ipilimumab
15	metastasis	≤1	none	75	metastasis	≤1	ipilimumab
16	metastasis	>1	ipilimumab	76	metastasis	≤1	ipilimumab
17	metastasis	≤1	none	77	metastasis	>1	ipilimumab
18	metastasis	≤1	none	78	metastasis	≤1	ipilimumab
19	primary	≤1	none	79	metastasis	≤1	ipilimumab
20	metastasis	>1	none	80	metastasis	≤1	ipilimumab
21	metastasis	≤1	none	81	metastasis	≤1	none
22	metastasis	≤1	none	82	metastasis	≤1	none
23	metastasis	>1	none	83	metastasis	≤1	none
24	primary	>1	none	84	metastasis	≤1	none
25	primary	>1	none	85	metastasis	≤1	none
26	metastasis	≤1	ipilimumab	86	metastasis	≤1	none
27	metastasis	≤1	ipilimumab	87	metastasis	≤1	none
28	metastasis	≤1	none	88	metastasis	≤1	none
29	metastasis	>1	ipilimumab	89	metastasis	≤1	none
30	metastasis	≤1	ipilimumab plus nivolumab	90	metastasis	≤1	none
31	metastasis	>1	ipilimumab	91	metastasis	≤1	none
32	metastasis	≤1	none	92	metastasis	≤1	none
33	metastasis	≤1	nivolumab	93	metastasis	≤1	none
34	metastasis	≤1	nivolumab	94	primary	≤1	none
35	metastasis	≤1	ipilimumab	95	metastasis	≤1	none
36	primary	>1	none	96	primary	≤1	none
37	metastasis	≤1	none	97	metastasis	≤1	none
38	primary	>1	none	98	metastasis	≤1	ipilimumab
39	metastasis	≤1	ipilimumab	99	primary	≤1	none
40	primary	>1	none	100	metastasis	≤1	none
41	metastasis	>1	atezolizumab	101	metastasis	≤1	nivolumab
42	metastasis	>1	none	102	metastasis	≤1	none
43	metastasis	≤1	none	103	metastasis	≤1	none
44	primary	≤1	none	104	metastasis	≤1	none
45	metastasis	>1	ipilimumab	105	metastasis	≤1	nivolumab
46	metastasis	≤1	none	106	metastasis	≤1	none
47	primary	≤1	none	107	metastasis	≤1	ipilimumab
48	metastasis	>1	ipilimumab	108	primary	>1	none
49	primary	>1	none	109	metastasis	≤1	ipilimumab
50	primary	≤1	none	110	metastasis	≤1	nivolumab
51	primary	≤1	none	111	metastasis	≤1	nivolumab
52	primary	>1	none	112	metastasis	≤1	none
53	metastasis	≤1	none	113	metastasis	≤1	none
54	metastasis	≤1	none	114	metastasis	≤1	none
55	primary	≤1	none	115	metastasis	≤1	none
56	metastasis	>1	ipilimumab	116	metastasis	>1	ipilimumab
57	metastasis	>1	none	117	primary	>1	none
58	metastasis	≤1	none				
59	metastasis	≤1	ipilimumab				
60	metastasis	≤1	none				

Supplementary Table 2. Univariable and multivariable Cox regression analyses for progression-free survival of melanoma patients and CAF parameters by quantitative immunofluorescence.

Variable (LO/HI)	Anti-PD-1 PFS					
	Univariable analysis		Multivariable* analysis per variable		Multivariable* analysis with Thy1 and FAP	
	HR (95% CI)	*P* value	HR (95% CI)	*P* value	HR (95% CI)	*P* value
$Thy1_{DAPI}$	1.50 (0.94–2.38)	0.087	**1.77 (1.08–2.93)**	**0.024**	1.40 (0.82–2.40)	0.22
SMA_{Thy1}	0.64 (0.38–1.15)	0.13	0.69 (0.39–1.30)	0.24		
FAP_{Thy1}	**1.92 (1.21–3.07)**	**0.0061**	**2.05 (1.27–3.31)**	**0.0031**	**1.81 (1.09–3.04)**	**0.023**

Abbreviations: CAF, cancer-associated fibroblast; CI, confidence interval; HI, high; HR, hazard ratio; LO, low.
*Cox proportional hazards model included age, sex, mutation status, stage, treatment, and prior immune checkpoint blockade as covariates.

Supplementary Table 3. Univariable and multivariable Cox regression analyses for overall survival of melanoma patients and CAF parameters by quantitative immunofluorescence.

Variable (LO/HI)	Control OS		Anti-PD-1 OS					
	Univariable analysis		Univariable analysis		Multivariable* analysis per variable		Multivariable* analysis with Thy1 and FAP	
	HR (95% CI)	*P* value	HR (95% CI)	*P* value	HR (95% CI)	*P* value	HR (95% CI)	*P* value
$Thy1_{DAPI}$	0.80 (0.52–1.25)	0.33	**2.38 (1.28–4.59)**	**0.0059**	**2.97 (1.53–6.01)**	**0.0011**	1.93 (0.94–4.11)	0.074
SMA_{Thy1}	0.58 (0.29–1.39)	0.20	0.54 (0.28–1.10)	0.089	0.63 (0.31–1.35)	0.23		
FAP_{Thy1}	**0.54 (0.35–0.82)**	**0.0038**	**3.53 (1.87–7.02)**	**<0.0001**	**3.63 (1.89–7.35)**	**<0.0001**	**2.81 (1.37–6.01)**	**0.0044**

Abbreviations: CAF, cancer-associated fibroblast; CI, confidence interval; HI, high; HR, hazard ratio; LO, low.
*Cox proportional hazards model included age, sex, mutation status, stage, treatment, and prior immune checkpoint blockade as covariates.

Supplementary Table 4. Univariable and multivariable Cox regression analyses for progression-free survival of anti-PD-1 treated melanoma patients and CAF parameters by cell counts per treatment group.

Variable (LO/HI)	Monotherapy (pembrolizumab or nivolumab) PFS			
	Univariable analysis		Multivariable* analysis per variable	
	HR (95% CI)	*P* value	HR (95% CI)	*P* value
Thy1$^+$/total	**2.93 (1.34–5.89)**	**0.0087**	**3.92 (1.52–9.60)**	**0.0058**
SMA$^+$/Thy1$^+$	0.81 (0.40–1.82)	0.59	0.70 (0.31–1.69)	0.41
FAP$^+$/Thy1$^+$	1.58 (0.85–2.99)	0.15	1.68 (0.89–3.22)	0.11
**Variable (LO/HI)**	**Dual therapy (ipilimumab plus nivolumab) PFS**			
	Univariable analysis		Multivariable* analysis per variable	
	HR (95% CI)	*P* value	HR (95% CI)	*P* value
Thy1$^+$/total	1.50 (0.44–3.84)	0.48	1.08 (0.29–3.09)	0.90
SMA$^+$/Thy1$^+$	**0.34 (0.16–0.79)**	**0.014**	**0.34 (0.14–0.88)**	**0.028**
FAP$^+$/Thy1$^+$	**2.21 (1.07–4.91)**	**0.032**	**2.29 (1.04–5.36)**	**0.040**

Abbreviations: CAF, cancer-associated fibroblast; CI, confidence interval; HI, high; HR, hazard ratio; LO, low; PFS, progression-free survival.
*Cox proportional hazards model included age, sex, mutation status, stage, and prior immune checkpoint blockade as covariates.

Supplementary Table 5. Univariable and multivariable Cox regression analyses for overall survival of anti-PD-1 treated melanoma patients and CAF parameters by cell counts per treatment group.

Variable (LO/HI)	Monotherapy (pembrolizumab or nivolumab) OS			
	Univariable analysis		Multivariable* analysis per variable	
	HR (95% CI)	*P* value	HR (95% CI)	*P* value
$Thy1^+$/total	**5.87 (2.41–13.58)**	**0.0002**	**8.55 (2.74–26.95)**	**0.0003**
SMA^+/$Thy1^+$	0.50 (0.21–1.30)	0.15	**0.21 (0.07–0.65)**	**0.0078**
FAP^+/$Thy1^+$	**6.59 (2.49–22.71)**	**<0.0001**	**6.18 (2.26–21.75)**	**0.0002**
Variable (LO/HI)	Dual therapy (ipilimumab plus nivolumab) OS			
	Univariable analysis		Multivariable* analysis per variable	
	HR (95% CI)	*P* value	HR (95% CI)	*P* value
$Thy1^+$/total	3.29 (0.93–9.23)	0.063	2.56 (0.64–8.52)	0.17
SMA^+/$Thy1^+$	**0.23 (0.09–0.65)**	**0.0067**	**0.28 (0.09–0.87)**	**0.028**
FAP^+/$Thy1^+$	2.42 (0.91–7.56)	0.077	2.44 (0.83–8.18)	0.11

Abbreviations: CAF, cancer-associated fibroblast; CI, confidence interval; HI, high; HR, hazard ratio; LO, low; OS, overall survival.

*Cox proportional hazards model included age, sex, mutation status, stage, and prior immune checkpoint blockade as covariates.
